# I22: SAXS/WAXS beamline at Diamond Light Source – an overview of 10 years operation

**DOI:** 10.1107/S1600577521002113

**Published:** 2021-03-15

**Authors:** A. J. Smith, S. G. Alcock, L. S. Davidson, J. H. Emmins, J. C. Hiller Bardsley, P. Holloway, M. Malfois, A. R. Marshall, C. L. Pizzey, S. E. Rogers, O. Shebanova, T. Snow, J. P. Sutter, E. P. Williams, N. J. Terrill

**Affiliations:** a Diamond Light Source Ltd, Diamond House, Harwell Science and Innovation Campus, Didcot, Oxfordshire OX11 0DE, United Kingdom; b ISIS Neutron and Muon Source, Science and Technology Facilities Council, Rutherford Appleton Laboratory, Didcot, Oxfordshire OX11 0QX, United Kingdom; c ALBA Synchrotron, Carrer de la Llum 2-26, 08290 Cerdanyola del Vallès, Barcelona, Spain; d King’s College London, Guy’s Campus, Great Maze Pond, London SE1 1UL, United Kingdom

**Keywords:** SAXS, WAXS, undulators, beamlines

## Abstract

Beamline I22 at Diamond Light Source is dedicated to the study of soft-matter systems from both biological and materials science. The beamline can operate in the range 3.7 keV to 22 keV for transmission SAXS and 14 keV to 20 keV for microfocus SAXS with beam sizes of 240 µm × 60 µm [full width half-maximum (FWHM) horizontal (H) × vertical (V)] at the sample for the main beamline, and approximately 10 µm × 10 µm for the dedicated microfocusing platform. There is a versatile sample platform for accommodating a range of facilities and user-developed sample environments. The high brilliance of the insertion device source on I22 allows structural investigation of materials under extreme environments (for example, fluid flow at high pressures and temperatures). I22 provides reliable access to millisecond data acquisition timescales, essential to understanding kinetic processes such as protein folding or structural evolution in polymers and colloids.

## Abstract 2

Beamline I22, a versatile SAXS/WAXS beamline at Diamond Light Source, is presented, along with an overview of 10 years operation of the beamline.

## 1. Introduction   

Small-angle X-ray scattering (SAXS) provides essential information on the structure and dynamics of large molecular assemblies in low-order environments. These are characteristic of living organisms and also many complex materials such as polymers and colloids. Relevant active research in the UK encompasses the fields of medicine (Ma *et al.*, 2016 ▸; Burton *et al.*, 2019 ▸; Coudrillier *et al.*, 2016 ▸; Al-Jaibaji *et al.*, 2018 ▸; Kudsiova *et al.*, 2019 ▸), biology (Troilo *et al.*, 2016 ▸; McGeehan *et al.*, 2011 ▸; Arnold *et al.*, 2011 ▸; Salamah *et al.*, 2018 ▸), the environment (Neill *et al.*, 2018 ▸; Seddon *et al.*, 2016 ▸) and materials (Summerton *et al.*, 2019 ▸; Wychowaniec *et al.*, 2018 ▸; Burton *et al.*, 2017 ▸), and includes studies of supramolecular organization in biomechanical systems (Xi *et al.*, 2018 ▸; Kampourakis *et al.*, 2018 ▸; Sui *et al.*, 2014 ▸), corneal transparency (Morgan *et al.*, 2018 ▸; Hayes *et al.*, 2017 ▸), biological membranes (Barriga *et al.*, 2016 ▸; Slatter *et al.*, 2018 ▸; Tang *et al.*, 2014 ▸), polymer processing (Stasiak *et al.*, 2015 ▸; Wan *et al.*, 2018 ▸; Heeley *et al.*, 2013 ▸; Toolan *et al.*, 2017 ▸), colloids (Calabrese *et al.*, 2019 ▸; Poulos *et al.*, 2016 ▸; Mable *et al.*, 2016 ▸), inorganic aggregates (Raine *et al.*, 2018 ▸; Bennett *et al.*, 2015 ▸; Zhou *et al.*, 2018 ▸), liquid crystals (Hallett *et al.*, 2014 ▸; Prehm *et al.*, 2018 ▸; Lehmann *et al.*, 2018 ▸) and devices (Xia *et al.*, 2018 ▸; Barrows *et al.*, 2016 ▸).

## 2. Beamline overview   

**Figure 1:**
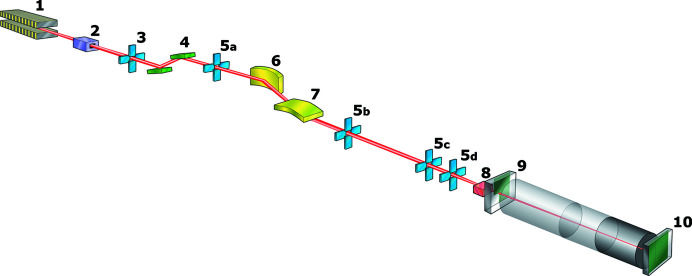
Layout of I22.

The scientific and technological challenges confronted by this diverse community required a high-resolution, high-brightness synchrotron beamline. The first small-angle scattering beamline at Diamond, I22 (see Fig. 1 ▸ and Table 1 ▸) uses an in-vacuum undulator source to deliver a high photon flux into a focused 240 µm × 60 µm spot [full width at half-maximum (FWHM), horizontal (H) × vertical (V)] at the sample for the main beamline, and approximately 10 µm × 10 µm for a dedicated microfocusing platform. This platform will be the subject of a further technical paper, and will not be discussed in detail here. The main beamline has the potential for the continuous energy range 3.7 keV to 22 keV with, at present, the reduced range 14 keV to 20 keV for the microfocus option. It is currently operated from 7 keV to 20 keV; lower-energy operation would require either helium or vacuum sample chambers. Depending on the energy used, and the exact geometry of the scattering experiment, the *q* range achievable at I22 is 0.0011 ≤ *q* (Å^−1^) ≤ 9.45. The SAXS camera is composed of evacuated sections of flight tube with a nosecone incorporating the WAXS detector at the sample end and ended by a 310 mm-diameter Kapton window at the SAXS detector end. These tubes can be connected as required to form camera lengths anywhere from 1.9 m to 9.9 m in 0.25 m steps. Changes to the configuration are manual so reconfiguration does take time, 20–60 min depending on camera length to be reconfigured. The vacuum achieved in the SAXS camera is 5 × 10^−5^ mbar. The primary endstation, with associated area detectors for static and time resolved measurements, is capable of recording the scattered radiation from samples contained in a range of commercial and bespoke sample environments.

## 3. Undulator   

**Figure 2:**
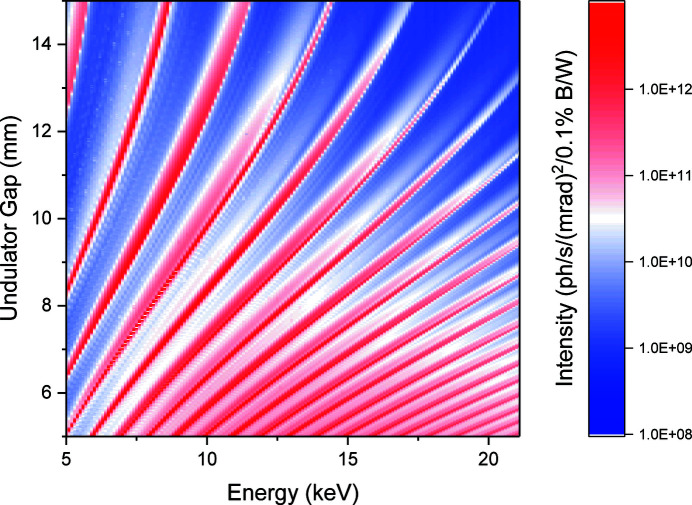
In-vacuum U25 undulator spectrum of I22. Flux values measured with calibrated diagnostic after the monochromator. NB: the primary white beam slits were closed to a central 100 µm × 100 µm gap to give a clean undulator spectrum. Flux and undulator gap values at selected typical energies are reported in Table 3 ▸.

The beamline operates a Diamond-designed (Patel *et al.*, 2017 ▸) 2 m in-vacuum undulator with a period of 25 mm, providing continuous energy coverage over the energy range of the beamline. The beamline is optimized for the energy range 8 keV to 20 keV working primarily with the 5th through to the 17th harmonics. Off-axis undulator radiation is removed to minimize heat load on the beamline optics via a water-cooled 150 µrad × 75 µrad (H × V) aperture located inside the storage ring tunnel 17.202 m downstream from the source. Water-cooled primary slits further define the beam before energy selection and focusing. The undulator has a very small phase error (0.02 mm). Fig. 2 ▸ shows the spectrum as a function of energy and undulator gap. All available harmonics can clearly be distinguished, from the 2nd at the top left, through to the 20th at the bottom right.

## 4. Monochromator   

The monochromator (Oxford Danfysik, Oxford, UK) is a fixed-exit double-crystal design, originally established for three crystal sets. It is currently installed with an Si(111) crystal pair; this allows access over the full operational energy range, with a resolution of 1.6 × 10^−4^ Δ*E*/*E* at 10.0 keV. Vertical translation of the second crystal maintains a fixed-beam exit configuration for the beamline. Both the first and the second crystals are indirectly cooled by liquid nitrogen to prevent damage arising from the high power density of the undulator. An indium foil provides thermal contact for both crystals to a cooled copper block. The first crystal is cooled by liquid nitrogen flowing continuously through the copper block while heat from the second crystal is removed via copper braiding. The Diamond storage ring has run in top-up mode since October 2008, with an injection every 10 min. This produces a very slight variation in ring current of ∼2% or ±5 mA at most; therefore, changes in the heat load on the monochromator during an experiment are primarily due to undulator gap and harmonic changes associated with a change in energy. Both coarse motors (±4 mrad) and fine piezo actuators (±180 µrad) are used to adjust the pitch and roll of the crystals, maintaining alignment of the first and second crystal lattice planes.

### 4.1. Monochromator calibration   

**Figure 3:**
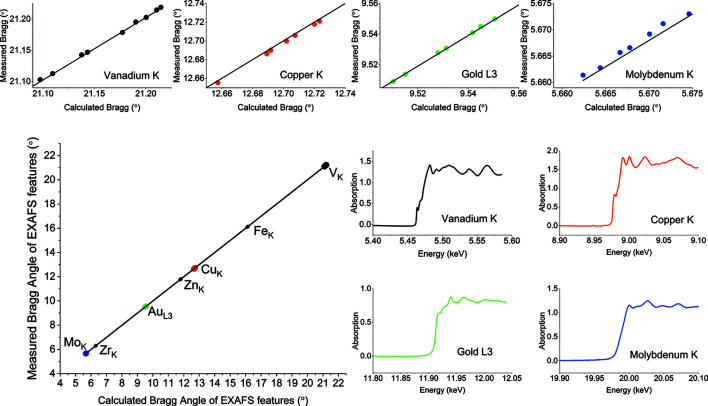
Calibration of the I22 monochromator using EXAFS from metal foils.

The monochromator is periodically calibrated using EXAFS spectra collected from a series of retractable metal foils that are permanently installed in the beamline downstream of the monochromator. Scatter diodes before and after the foils record *I*_0_ and *I*_*t*_ values, respectively. EXAFS features from the spectra (peaks), and the derivative of the spectra (edges) for all of the foils (V, Fe, Cu, Zn, Au, Zr, and Mo), are fitted, against their expected positions from literature values (Bearden & Burr, 1967 ▸), in a global least-squares minimization. The main part (lower left) of Fig. 3 ▸ shows the overall fit. The lower right section of Fig. 3 ▸ shows the absorption spectra from V, Cu, Au and Mo as measured on I22. Expanded views of the linear fitting of the vanadium, copper, gold and molybdenum features are shown along the top, demonstrating the consistency of the fit across a wide range of Bragg angles. In a typical fit, 46 data points spanning 15.55° are used to calibrate the Bragg axis.

As a means of evaluating the repeatability, ten spectra were measured for each of the above edges. Data are provided in the supporting information. Standard deviations for the measured positions of EXAFS features of 3.8 µrad at the vanadium edge and 1.8 µrad at the molybdenum edge were found, highlighting the excellent repeatability of the monochromator design.

## 5. Mirrors   

The beamline focusing comprises a Kirkpatrick–Baez (KB) (Kirkpatrick & Baez, 1948 ▸) mirror pair (ACCEL, Germany), using adaptive bimorph mirror technology (Susini *et al.*, 1995 ▸; Cautero *et al.*, 2007 ▸) to provide independent horizontal and vertical focusing at any point in the endstation while maintaining operation close to 1:1 focusing. Bimorph mirrors have three advantages that make them useful for I22. First, by varying the voltages on individual electrodes, one can correct the waviness left on the mirror surface by polishing, thus achieving a sharp focal spot with small tails. Second, a bimorph responds within seconds to changes in voltage, and even settling times imposed by imperfect mounting of the bimorph generally do not exceed 10–15 min. Therefore, bimorph mirrors permit the size, shape and focal distance of the X-ray beam to be changed rapidly to accommodate different camera lengths. Third, they have good long-term stability, and thus can be left at a constant voltage setting over weeks or months without loss of focal quality. The horizontal focusing mirror (900 mm long, with an 820 mm active length) and the vertical focusing mirror (600 mm long, with a 550 mm active length) are located at 26.8 m and 27.7 m, respectively, from the source and share a common vacuum vessel. Each mirror substrate is made of fused silica, 50 mm-wide with a central 15 mm-wide coated stripe of rhodium; the stripes provide excellent harmonic rejection across the beamline energy range while operated at 2.6 mrad. The adaptive bimorph capability is provided by piezo actuators attached to each substrate. The vertical mirror has 32 electrodes, which are paired along the length to give 16 virtual electrodes. These are used to correct the mirror figure and provide vertical focusing. The horizontal mirror has 12 electrodes, 4 at either end are coupled into 2 pairs and the 4 remaining electrodes are left as singles to give finer control over the central portion of the mirror, where it is of greatest benefit for providing optimal horizontal focusing.

### 5.1. Mirror refurbishment   

**Figure 4:**
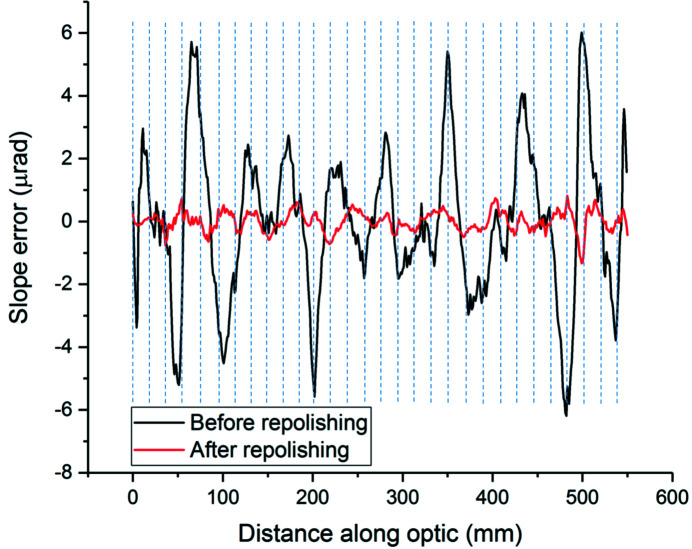
Effect of repolishing the I22 VFM as determined by Diamond NOM.

Examination of the bimorph mirrors using the X-ray beam and the Diamond Light Source nanometre optical metrology (NOM) (Alcock *et al.*, 2010 ▸) non-contact profiler revealed damage at the piezo interfaces (Alcock *et al.*, 2013 ▸) as indicated by the gross changes in slope error across the mirror in the measurements shown in Fig. 4 ▸. Although not all junctions behaved the same (dotted lines in Fig. 4 ▸ show the position of the junctions, and features are invariably found near the junctions), these effects translated into poor focusing performance, rendering optimum focusing impossible and causing degradation in beamline background. Repolishing the optical surface of both mirrors significantly improved their focusing capability to the point where optimum focus was achievable. Data from the Diamond NOM shows a ninefold improvement in slope error after repolishing from 2.7 µrad to 0.3 µrad.

We stress that the junction effect was not found to appear in the latest ‘second-generation’ bimorph mirrors, which have their piezoelectric plates attached to the sides of the mirror substrate rather than being sandwiched between an upper and a lower substrate, as is the case with the I22 first-generation bimorph mirrors (Alcock *et al.*, 2019*a* ▸,*b* ▸).

Table 2 ▸ gives an indication of the beam size at various camera lengths on I22 after correcting the mirror surfaces. These measurements do not perfectly match the theoretical widths. However, with reasonable values of electron beam size and mirror slope error, the theoretical widths come within 16% of the measured horizontal widths, and 10% of the measured vertical widths (Table 2 ▸).

## 6. Endstation   

**Figure 5:**
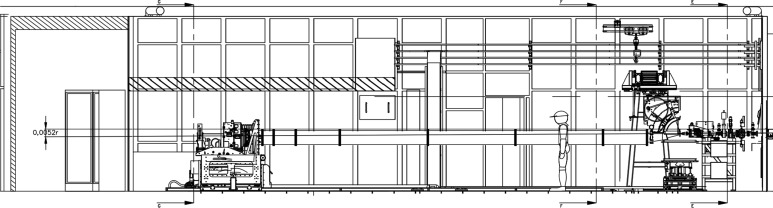
Schematic representation of the I22 endstation.

The endstation on I22 is a versatile space, with a large sample platform that can accommodate a wide range of sample environments. Fig. 5 ▸ shows a schematic view of the space, and Fig. S5 of the supporting information shows two sample enviroments mounted side-by-side on the table in a typical experimental configuration.

### 6.1. Flux calibration   

Flux values are reported in Table 3 ▸ for a selection of commonly used experimental conditions. The measurements were taken using a calibrated diode (Canbera PD-300 PIPS diode calibrated by PTB Berlin) at the sample position.

### 6.2. Sample environments   

Samples are mounted on a platform (IDT, Cheshire, UK; customized design) allowing remotely controlled motion of 200 mm both horizontally and vertically. The platform is mounted on rails for manually controlled motion of 1000 mm along the beam path. Combined with motorized *X*, *Y* and manual *Z* motion of the WAXS detector nosecone, any reasonable size sample environment can be accommodated whilst minimizing air gaps and attendant background scatter. The sample table surface comprises a standard optical breadboard (750 mm × 750 mm, M6 tapped holes on a 25 mm pitch) which facilitates mounting of sample environments, large and small. The platform has 5 µm resolution, and can accommodate large sample environments (up to 100 kg) provided either in-house or built by users. If a small sample environment is required (up to 2 kg), such as a single sample cell or lightweight capillary holder, two motorized translation stages equipped with stepper motors (Newport Spectra-Physics Ltd, Didcot, UK; UTS100PP) provide precise sample positioning with 1 µm resolution over a 100 mm range of motion horizontally and vertically. The usual sample environments found on versatile SAXS beamlines are also available on I22. These can be controlled remotely and can trigger – or be triggered – by the data acquisition software. Both Linkam DSC and Capillary furnaces (Linkam Scientific, Surrey, UK) are available for use accessing temperatures in the range −180°C to 550°C. Two commercial stopped-flow apparatus (SM 400; Bio-Logic, Grenoble, France) specifically designed for synchrotron radiation SAXS are available. One is used for investigations of conformational changes in proteins (Panine *et al.*, 2006 ▸), nucleic acids and macromolecules, while the second is dedicated to materials science studies (Lund *et al.*, 2013 ▸). A stress-controlled rheometer (Physica MCR 501; Anton Paar, Hertford, UK) is available with a range of geometries, suitable for a wide range of viscosities ranging from water to polymer melts (Poulin *et al.*, 2016 ▸). It can be temperature controlled using air or water, providing an operating range between −20°C and 350°C. A micromechanical tensile tester has been developed in collaboration with Queen Mary University of London (Karunaratne *et al.*, 2012 ▸). This can be used in tension, compression and bending modes. A millisecond pressure-jump cell (1 to 5000 bar) for use in X-ray scattering experiments has been developed in collaboration with Imperial College London, and is now routinely available on I22 (Brooks *et al.*, 2010 ▸). For higher pressure, a diamond anvil cell is available for use with the microfocus beam (Almax-easyLab, Diksmuide, Belgium; Boehler-Almax PlateDAC). This cell covers the pressure range 1 GPa to 60 GPa.

### 6.3. Detectors   

**Figure 6:**
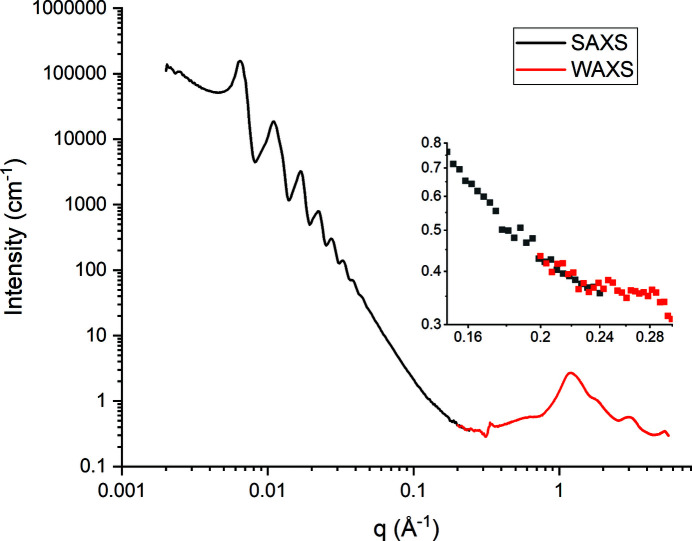
Overlapping *q*-scale of I22 detectors at 9 m camera length, the SAXS *q*-scale is 0.002–0.24 Å^−1^, the WAXS *q*-scale is 0.2–5.6 Å^−1^. Data are from 100 nm silica spheres collected as a powder between two Scotch Tape windows. NB: the inset shows the overlap region in more detail.

The beamline has a matched pair of Dectris (Dectris AG, Switzerland) Pilatus P3 Hybrid silicon pixel detectors (Henrich *et al.*, 2009 ▸), see Table 4 ▸. Of particular note is the in-vacuum L-shaped WAXS detector. This has the basic form of a Pilatus P3-2M with three modules in the lower right corner removed. The detector is built into the vacuum space of the SAXS camera nosecone, affording partial 2D access to WAXS to complement full 2D SAXS in the appropriate configuration. To give the reader an idea of the type of data coverage available in this configuration, silver behenate (a common SAXS standard) has been included in Fig. S4 in the supporting information. With careful beamline set-up, these detectors allow overlap of 1D SAXS and WAXS data over the full camera length capability of the beamline as highlighted by the data shown in Fig 6 ▸. The data shown were collected at 12.4 keV with a single 100 ms frame for both SAXS and WAXS detectors. It is possible to arrange the detectors such that no shadowing of the SAXS detector by the WAXS detector occurs.

## 7. Software   

The beamline is controlled using an architecture based on the *Experimental Physics and Industrial Control System* (*EPICS*) (Dalesio *et al.*, 1994 ▸). A graphical synoptic of the whole beamline is available and windows for specific components can be accessed to set and monitor process variables. Users interact with the beamline through the generic data acquisition client (GDA) (Rees *et al.*, 2010 ▸; Gibbons *et al.*, 2011 ▸), a software-based data acquisition system that sits on top of the *EPICS* layer. For simple data collection, frames × collection length, there are a range of graphical user interfaces available, and for more complex experiments the full Jython scripting capability of GDA can be employed.

After the acquisition of frame data, by default, GDA triggers any requested analyses that are available through the *Data Analysis WorkbeNch* (*DAWN*) software package (Basham *et al.*, 2015 ▸; Filik *et al.*, 2017 ▸). Most commonly, this analysis entails the reduction of frame data to a one-dimensional dataset of intensity (*I*) plotted against the scattering wavevector (*q*) following a standardized schema (Pauw *et al.*, 2017 ▸). It is also possible to analyse particular Debye–Scherrer rings, producing *I* versus azimuthal angle, χ, plots, the calculation of orientation factors and degrees of orientation (Cinader & Burghardt, 1998 ▸; Hermans, 1944 ▸) as well as crystallite thickness information from frame data (Fratzl *et al.*, 1996 ▸). These scattering-specific analyses are presented alongside a number of other common analytical methodologies, such as function fitting, any number of which can be automatically triggered following data acquisition.

## 8. Science examples   

From its inception, I22 was designed to accommodate the wide-ranging scientific requirements of the UK SAXS community. This covers most areas of science from biology and medicine through to advanced materials (O’Sullivan *et al.*, 2011 ▸; Inamdar *et al.*, 2017 ▸; Tang *et al.*, 2014 ▸; Brady *et al.*, 2019 ▸; Bots *et al.*, 2014 ▸; Besselink *et al.*, 2016 ▸; Lutz-Bueno *et al.*, 2016 ▸). It has successfully shown its flexibility by accommodating a wide array of diverse sample environments during its years of operation. Some of the more extreme examples have included a complete gold sputter chamber including a full manipulation system, chamber and local computer control hardware for exploring thin-film structure formation (Roth *et al.*, 2015 ▸) and a chemical reactor for investigating anoxic calcium carbonate formation (Ahmed *et al.*, 2010 ▸). The I22 sample environment portfolio includes many of the standard sample environments you would expect to see at a SAXS/WAXS beamline such as Linkam capillary (Summerton *et al.*, 2018 ▸) and DSC furnaces (Castelletto *et al.*, 2018 ▸), stop flow devices (Angelov *et al.*, 2011 ▸) and commercial rheometers (Wychowaniec *et al.*, 2020 ▸).

**Figure 7:**
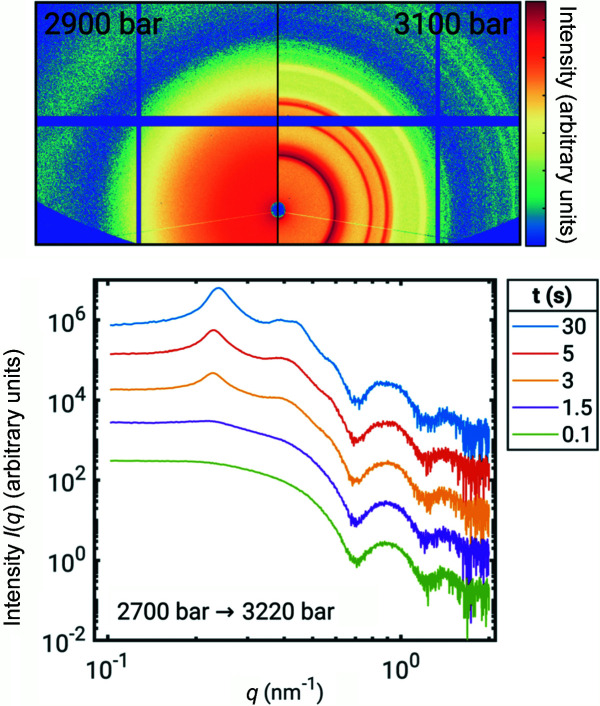
Dynamics of gold nanoparticle supercrystal formation. Republished with permission of Royal Society of Chemistry, from Lehmkühler *et al.* (2019 ▸), *Phys. Chem. Chem. Phys.* **21**, 21349–21354; permission conveyed through Copyright Clearance Center, Inc.

Of particular note in this area is the P-jump cell developed in collaboration with Imperial College (Brooks *et al.*, 2010 ▸). Due to the collaborative development of the cell its functionality has been fully embedded into the beamline control system which has enabled an integrated approach to data collection from the outset. The cell has been in successful operation since 2011 delivering across a range of scientific areas (Bots *et al.*, 2012 ▸; Carriero *et al.*, 2014 ▸; Balmer *et al.*, 2011 ▸; McCarthy *et al.*, 2017 ▸; Lehmkühler *et al.*, 2019 ▸; Fox *et al.*, 2020 ▸) covered by the beamline. We believe that the millisecond pressure jump capability of the cell is unique. An example of exploitation of this capability can be found in a recently published paper on the formation of gold nanoparticle super crystals (Lehmkühler *et al.*, 2019 ▸). Utilizing the fast P-jump nature of the cell they were able to demonstrate pressure-induced formation of super crystals from PEGylated gold nanoparticles (see Fig. 7 ▸). By varying the pressure, different nanostructures could be prepared.

**Figure 8:**
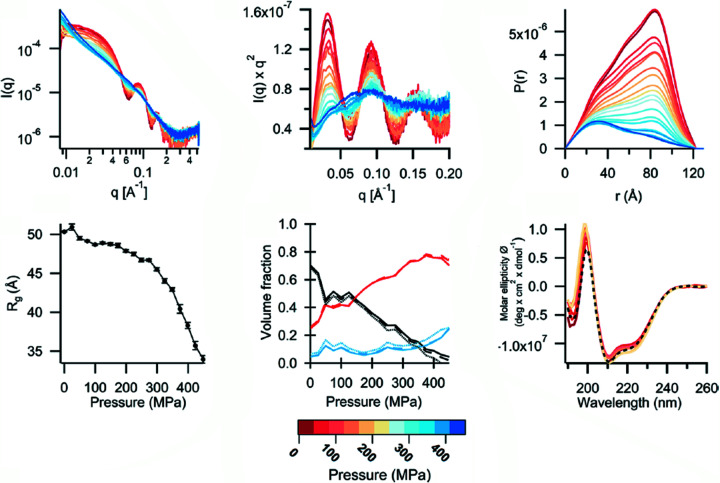
Disassembly/reassembly of bacterioferritin using pressure. Reprinted (adapted) with permission from Le Vay *et al.* (2020 ▸), *J. Am. Chem. Soc.* **142**, 20640–20650. Copyright (2020).

A paper published recently highlighted the versatility of the cell for studying complex systems. Le Vay *et al.* (2020 ▸) studied the controlled assembly and disassembly of nanoscale protein cages with a view to being able to use the system for capturing active ingredients upon reassembly (see Fig. 8 ▸).

**Figure 9:**
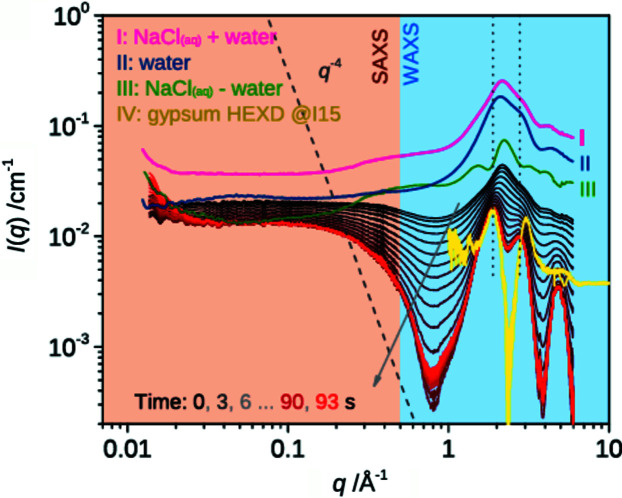
Bassanite crystallization kinetics, highlighting the full *q*-range coverage on I22. Reprinted (adapted) with permission from Stawski *et al.* (2019 ▸), *J. Phys. Chem. C*, **124**, 8411–8422. Copyright (2019) American Chemical Society.

The unique combination of a matched pair of Pilatus silicon hybrid pixel detectors for collecting both SAXS and WAXS data has also proven useful. Due to the design of the WAXS detector the beamline can collect high-quality SAXS and WAXS data simultaneously over a large solid angle. This has been exemplified by a recent paper on the nucleation pathways of bassanite by Stawski *et al.* (2020 ▸) (see Fig. 9 ▸). The overlapping nature of the design, described in Section 6.3, afforded the user group to clearly identify the phases of interest.

## 9. Summary   

I22 was designed as a multipurpose, undulator driven, combined SAXS–WAXS facility to serve the needs of a broad UK community of SAXS/WAXS users. Therefore, the beamline was designed as a very versatile platform capable of accommodating a wide variety of biological and soft-matter studies. The range of science covered has grown during its operation to include users supported by all of the major UK Science Research Councils as well as European funding bodies and beyond. Being the primary multipurpose SAXS beamline of Diamond, it has been required to carry out work ranging from studying precursors to life on earth (Tang *et al.*, 2014 ▸), through self-assembly of polymer thin films (Pearson *et al.*, 2014 ▸), to microfocus SAXS mapping studies on bone (Karunaratne *et al.*, 2013 ▸) and cornea for a range of medical studies (*e.g.* Hayes *et al.*, 2013 ▸). The selected examples illustrate the potential of the I22 beamline as a high-flux, high-resolution X-ray scattering beamline for *in situ* and time resolved investigations.

## Supplementary Material

Supporting information: Section S1: Calibration EXAFS spectra and derivatives; Section S2: GDA Perspectives used by I22; Section S3: Endstation and sample environments. DOI: 10.1107/S1600577521002113/ig5095sup1.pdf

## Figures and Tables

**Table 1:** Major components of I22

Fig. 1 ▸ index	Component	Distance from source (m)
1	In-vacuum undulator 25 mm period	0.00
2	Customized aperture	17.20
3	White beam slits	22.70
4	Double-crystal monochromator (Si 111)	24.00
5a–5d	Monochromatic slits	25.57, 28.55, 46.00, 47.00
6	Horizontal focusing mirror	26.78
7	Vertical focusing mirror	27.73
8	Sample position	47.1 to 48
9	Wide-angle detector	0.17 (from sample)
10	Small-angle detector	1.9 to 9.9 (from sample)

**Table 2:** Beam sizes for the repolished bimorph mirrors at 12.4 keV

Focal position	Demagnified FWHM source size (H × V, µm)	H FWHM slope error broadening (µm)	V FWHM slope error broadening (µm)	Measured FWHM focal size (H × V, µm)
Sample	258 × 6	39	60	262 × 60
Sample + 3 m	296 × 7	45	69	299 × 69
Sample + 5 m	321 × 7	49	75	329 × 75
Sample + 7 m	346 × 8	52	81	350 × 81

**Table 3:** Measured flux, undulator harmonic and undulator gap for commonly used energies

Energy (keV)	Measured flux (photons s^−1^)	Undulator harmonic	Undulator gap (mm)
7	1.08 × 10^13^	5th	7.035
10	6.94 × 10^12^	7th	7.160
12.4	3.97 × 10^12^	9th	6.921
14	2.74 × 10^12^	11th	6.420
18	1.48 × 10^12^	17th	5.359

**Table 4:** Detectors available on I22 and their main uses

Detector	Use
Pilatus P3-2M	(Gi)SAXS data collection
	172 µm pixel size
	Total active area 254 mm × 289 mm
	Frame rate 250 Hz with 1 ms readout
Pilatus P3-2M-DLS-L	In-vacuum (Gi)WAXS data collection
	172 µm pixel size
	Total active area 254 mm × 289 mm
	Frame rate 250 Hz with 1 ms readout
Vortex single element	Fluorescence detection for mapping experiments
Silicon pin diode	Absorption correction data collection
	1 mm^2^ diode embedded in beamstop
