# Supplementary material for: I22: SAXS/WAXS beamline at Diamond Light Source – an overview of 10 years operation
Source: J Synchrotron Radiat. 2021 Mar 15;28(Pt 3):939–47. doi: 10.1107/S1600577521002113 (PMC8127364; doi:10.1107/S1600577521002113)
Supplement: Supplementary file 1 [file s-28-00939-sup1.pdf]

JOURNAL OF SYNCHROTRON RADIATION

Volume 28 (2021)

Supporting information for article:

I22: SAXS/WAXS beamline at Diamond Light Source – an overview of 10 years operation

A. J. Smith, S. G. Alcock, L. S. Davidson, J. H. Emmins, J. C. Hiller Bardsley, P. Holloway, M. Malfois, A. R. Marshall, C. L. Pizzey, S. E. Rogers, O. Shebanova, T. Snow, J. P. Sutter, E. P. Williams and N. J. Terrill

Supplementary Information - I22: SAXS/WAXS beamline at Diamond Light Source - an overview of 10 years operation

A. J. Smith,[a] S. G. Alcock,[a] L. S. Davidson,[a] J. H. Emmins,[a] J. C. Hiller Bardsley,[d] P. Holloway,[a] M. Malfois,[c] A. R. Marshall,[a] C. L. Pizzey,[a] S. E. Rogers,[b] O. Shebanova,[a] T. Snow,[a] J. P. Sutter,[a] E. P. Williams[a] and N. J. Terrill [a]*

[a]*Diamond Light Source Ltd, Diamond House, Harwell Science and Innovation Campus, Didcot, Oxfordshire, OX11 0DE, United Kingdom,* [b]*ISIS Neutron and Muon Source, Science and Technology Facilities Council, Rutherford Appleton Laboratory, Didcot, Oxfordshire, OX11 0QX, United Kingdom,* [c]*ALBA Synchrotron, Carrer de la Llum 2-26, 08290 Cerdanyola del Vallès, Barcelona, Spain, and* [d]*King's College London, Guy's Campus, Great Maze Pond, London SE1 1UL United Kingdom. E-mail: nick.terrill@diamond.ac.uk*

1. Calibration EXAFS spectra and derivatives

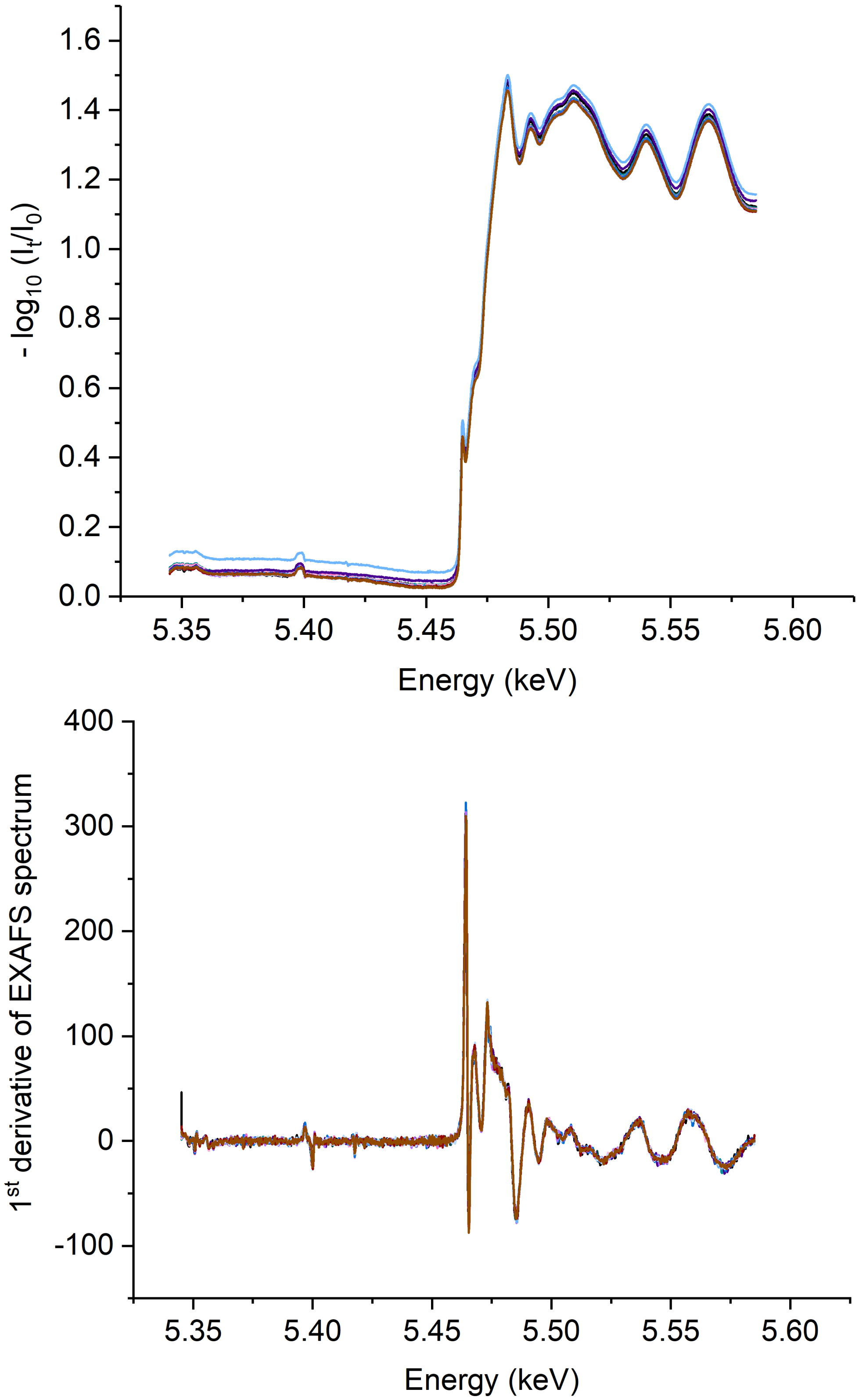

IUCr macros version 2.1.10: 2016/01/28

Fig. S1. Vanadium K edge EXAFS @ 0.2 eV resolution

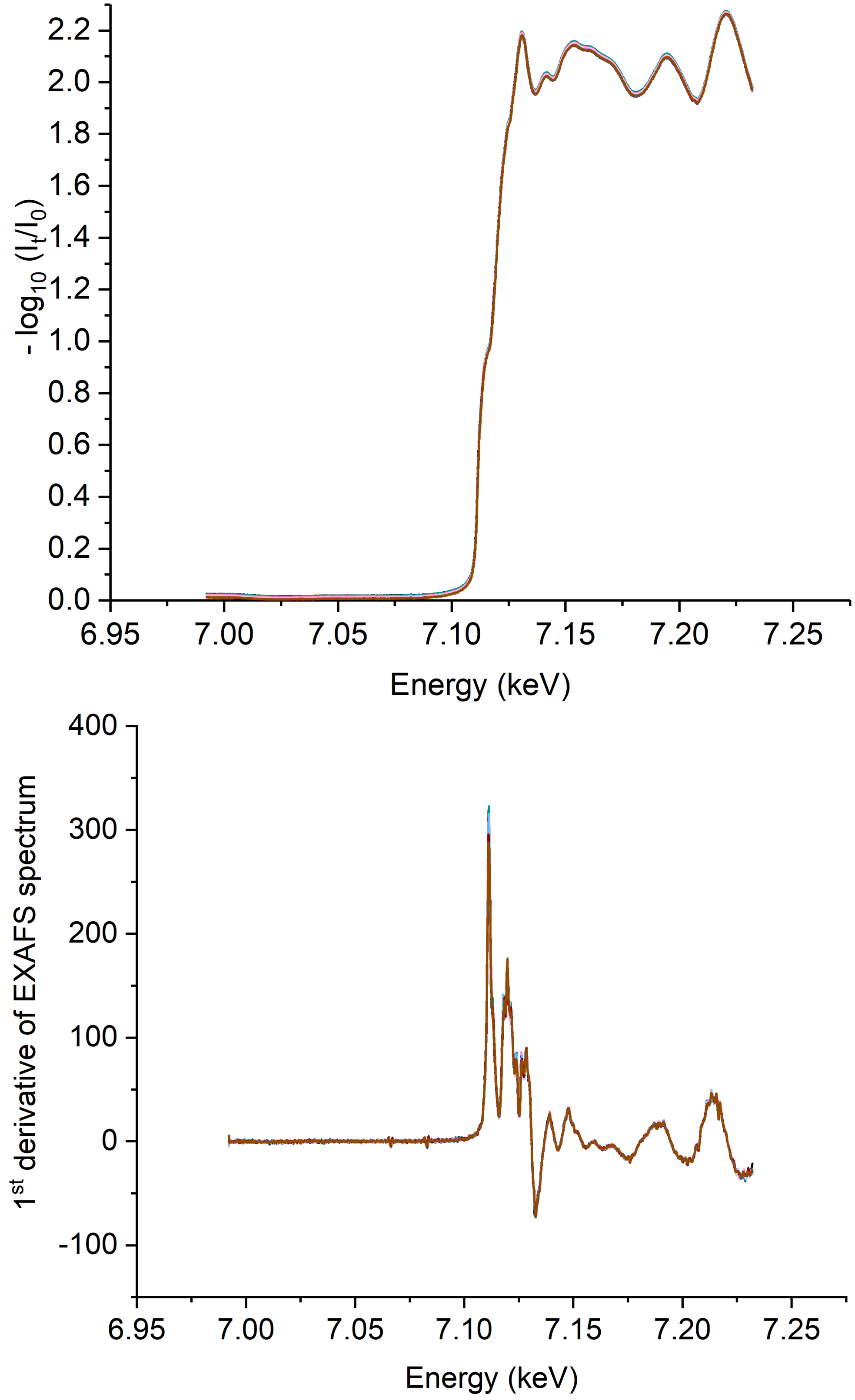

Fig. S2. Iron K edge EXAFS @ 0.2 eV resolution

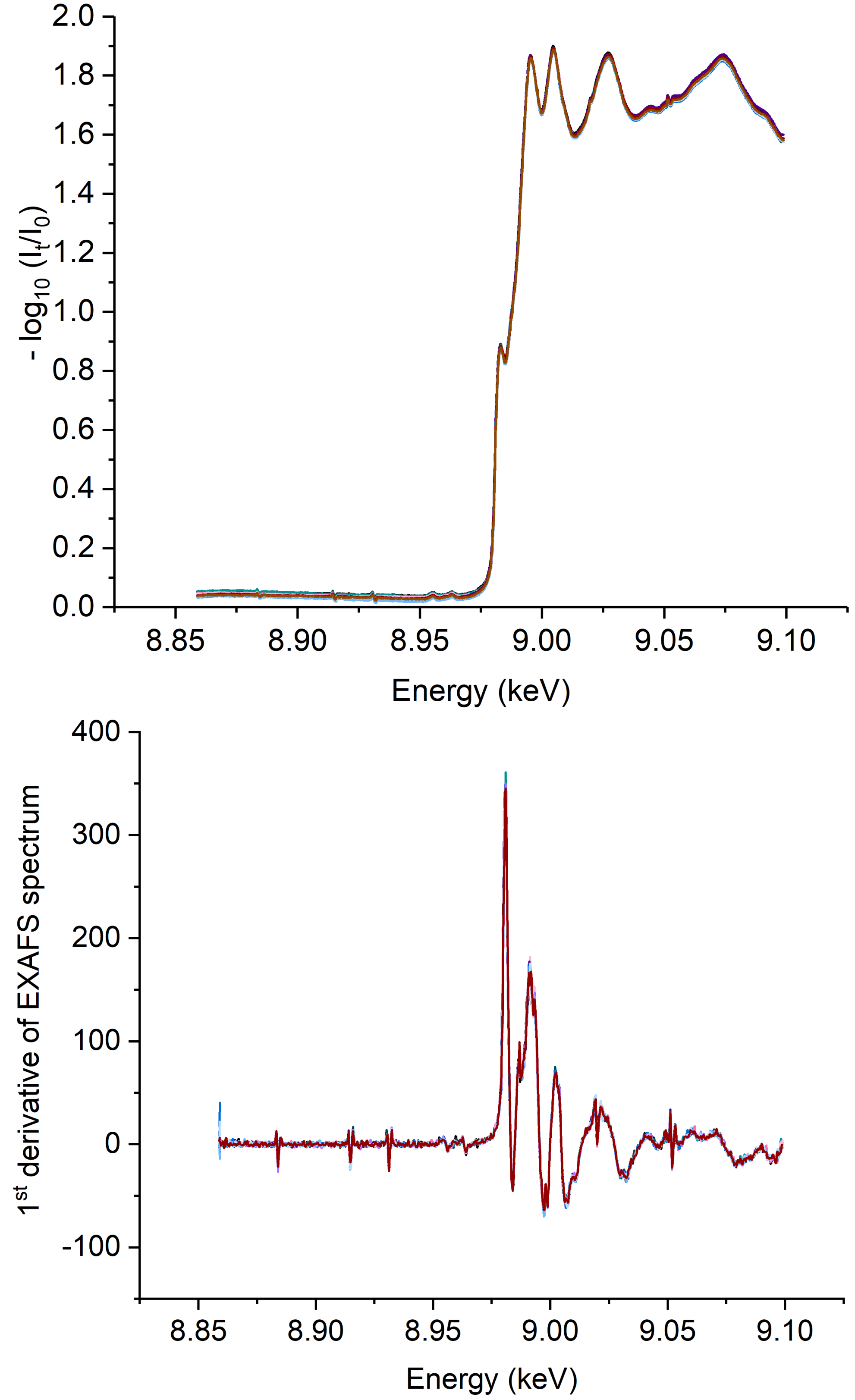

IUCr macros version 2.1.10: 2016/01/28

Fig. S3. Copper K edge EXAFS @ 0.2 eV resolution

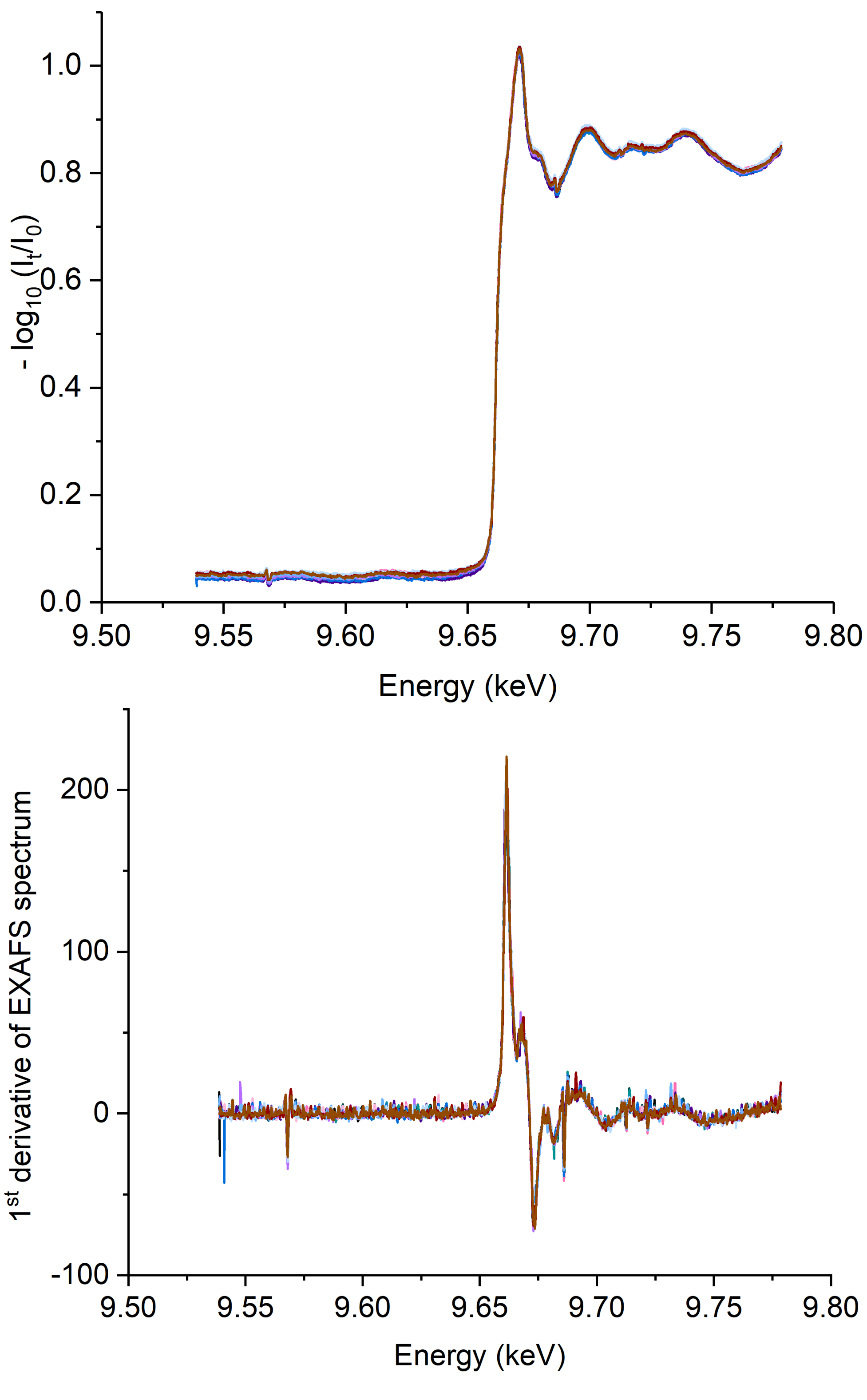

Fig. S4. Zinc K edge EXAFS @ 0.2 eV resolution

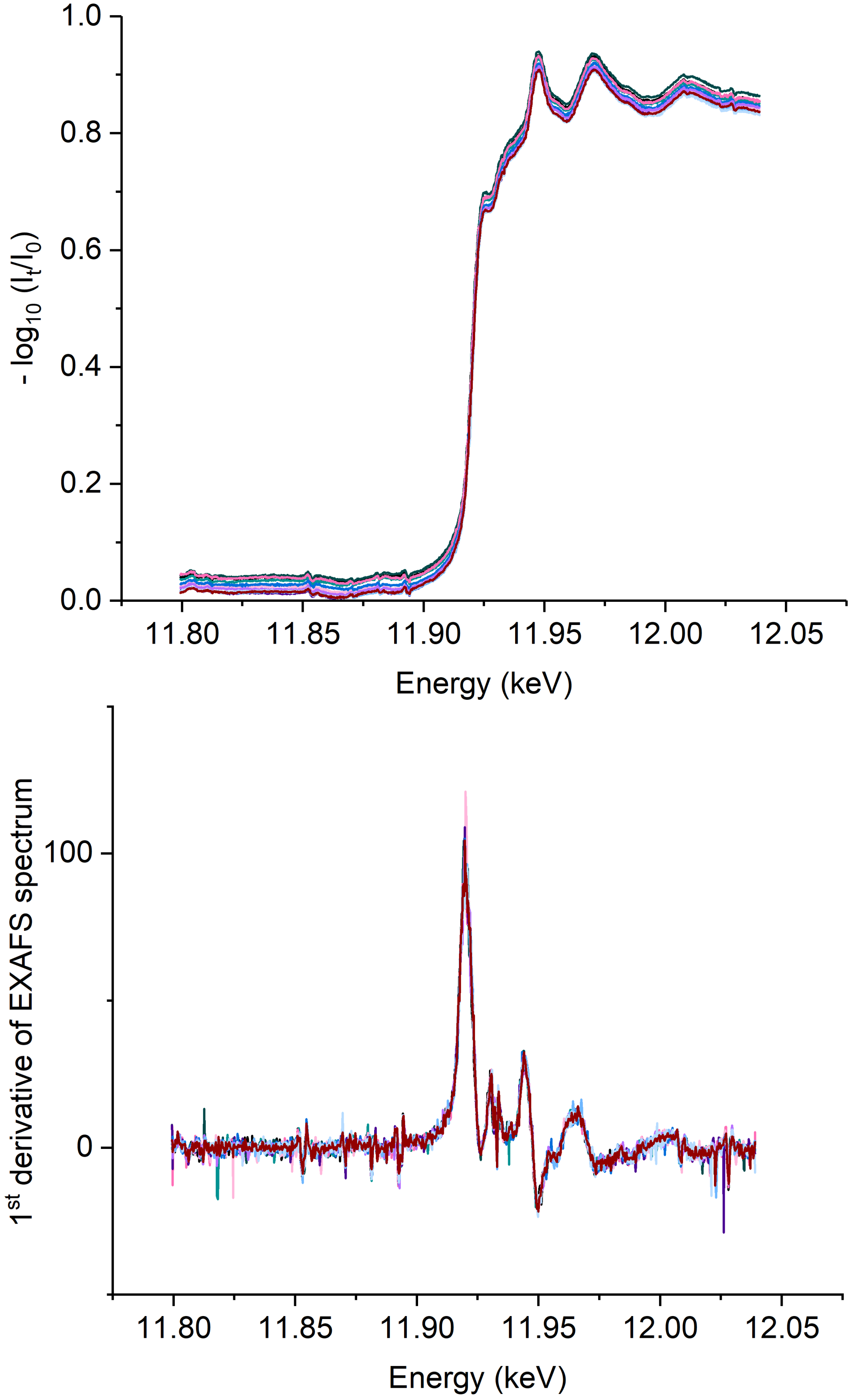

Fig. S5: Gold LIII edge EXAFS @ 0.2 eV resolution

IUCr macros version 2.1.10: 2016/01/28

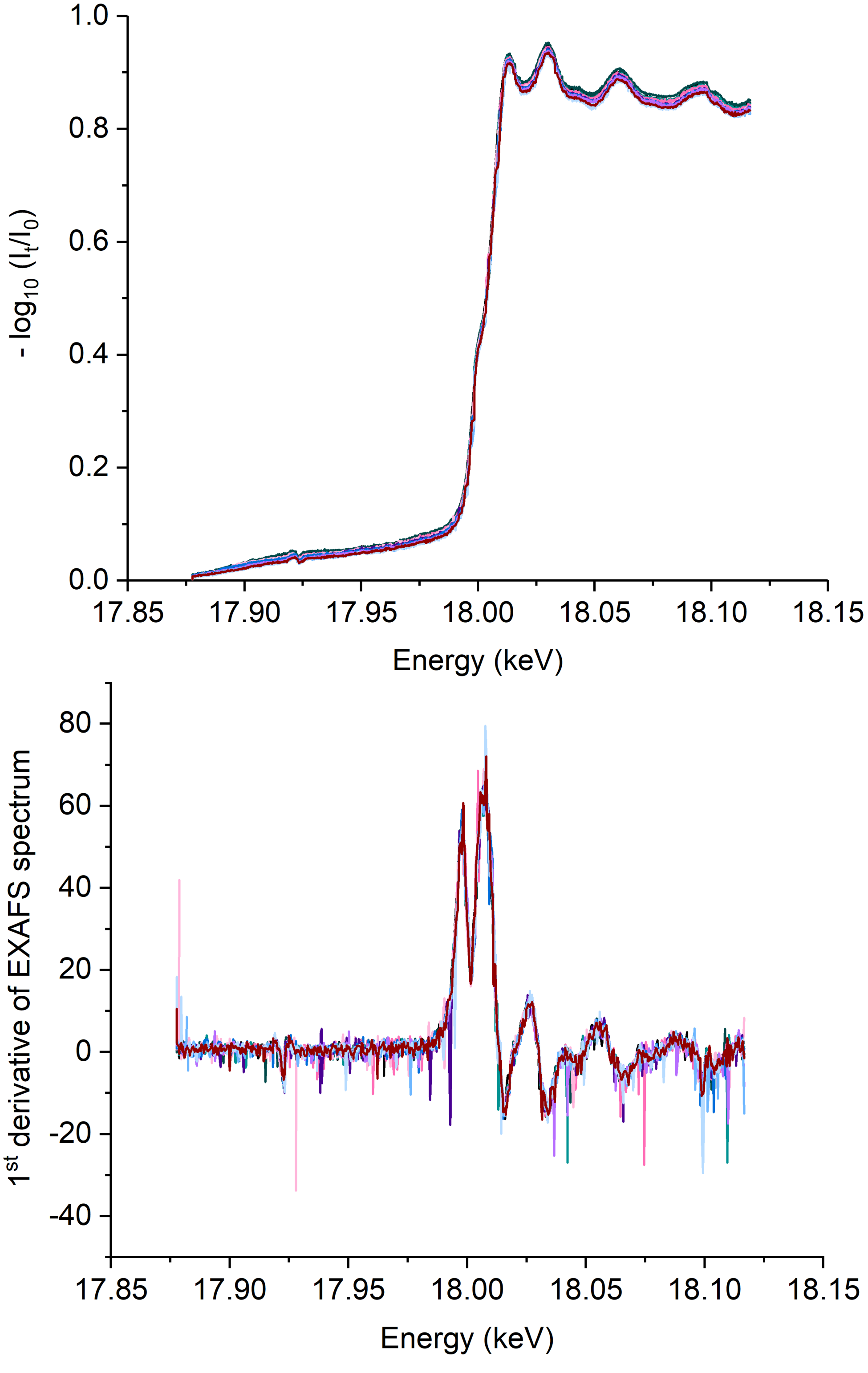

Fig. S6. Zirconium K edge EXAFS @ 0.2 eV resolution

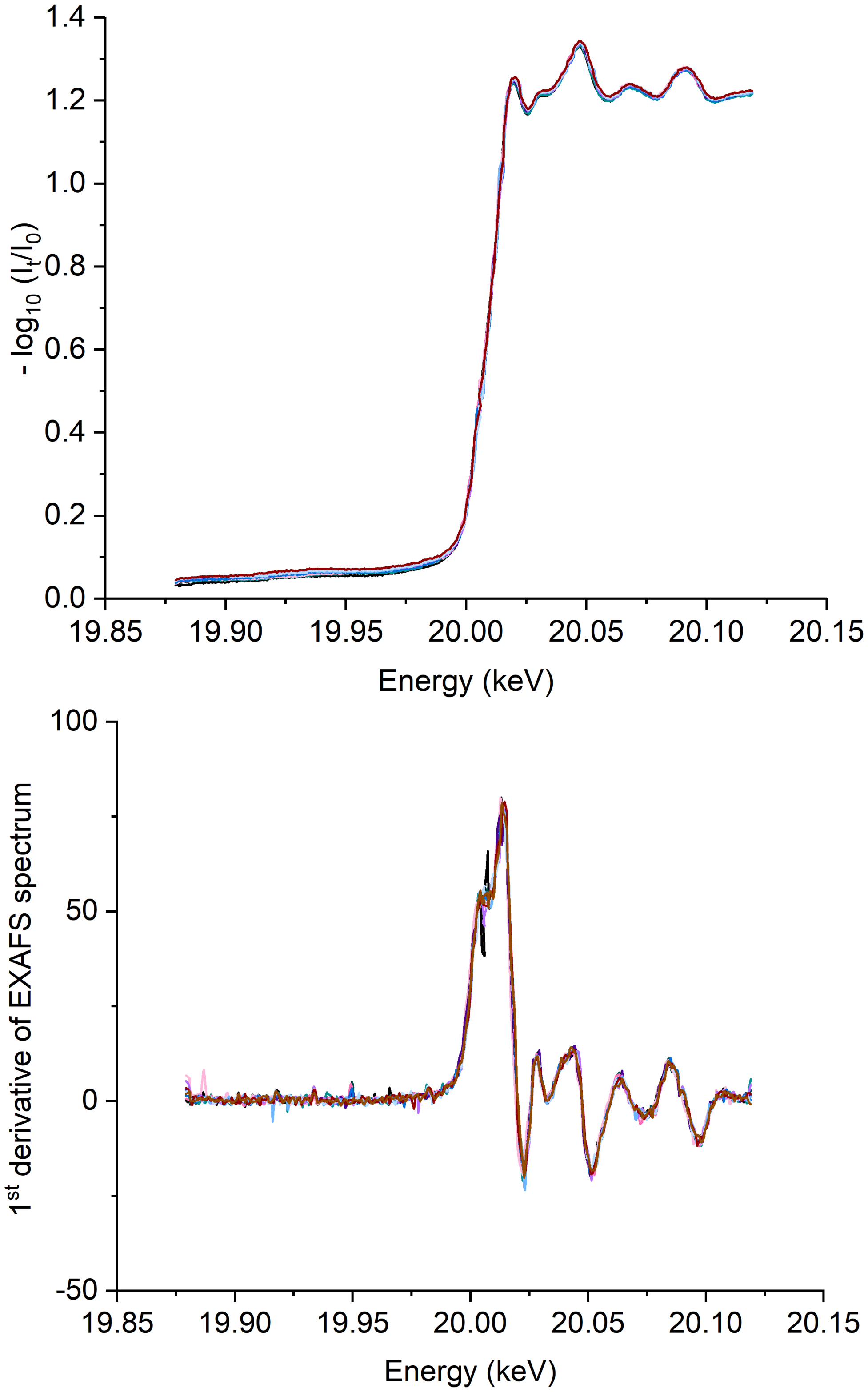

Fig. S7. Molybdenum K edge EXAFS @ 0.5 eV resolution

2. GDA Perspectives used by I22

I22 uses a series of GDA perspectives to support data collection on the beamline. GDA is, at its simplest, a scripting framework for collecting data. The simplest perspective is an interface for developing scripts for data collection. The figure depicts a capillary ladder script which will position samples in the beam, collect the data and save it with metadata such as sample name and thickness for later processing.

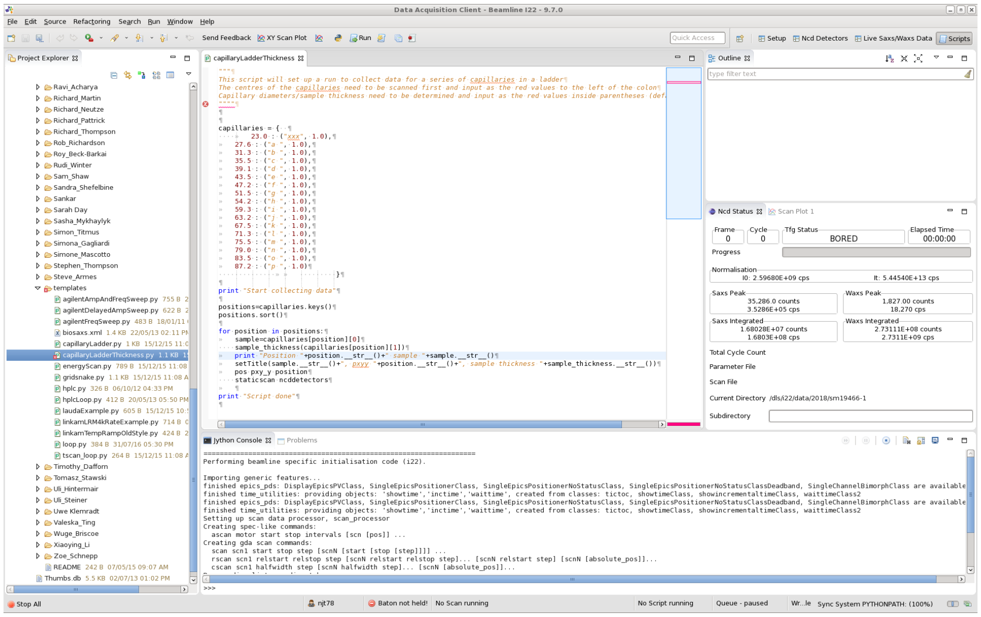

Fig. S1. Scripting Perspective with script for capillary ladder

The generic Setup Perspective is for running simple scan commands to control motors and other beamline components in the Jython Console; monitor, via the Polling Dashboard panel, diodes and other experimental PV's help set up the basic experimental configuration. It will also visualize the data from these scans in the Plot windows, e.g. sample cell alignment; and receive an overview of the progress of experiments when running via the NCD Status screen.

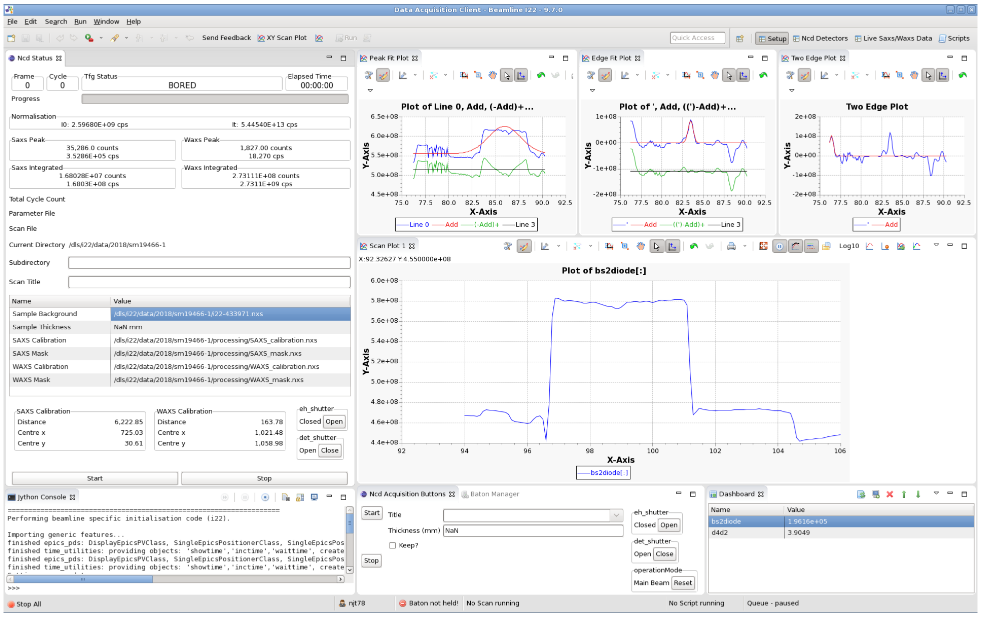

Fig. S2. Setup Perspective

The NCD Detectors Perspective has been developed to allow the user to set up the parameters that configure the x-ray detectors for data collection. The NCD Status tab gives an overview of progress through a data collection run. It also displays the name of the last saved data file and its location together with the current calibration information. The NCD Detector Settings tab gives access to configuring the resolution of the NCD detector. If this function is available for a given detector you will be able to change the "binning" of that detector. The Calibration Labels tab allows you to set up a ready reference table for the various scalars that are being saved with your data. The Time Frame Generator tab allows you to set up the length, number and triggering for time framed x-ray data collection. If you make changes here you must configure the TFG before the changes will take affect using the "Configure" button at the bottom of the tab. You can also save configuration for future use using the "Save" button and load them latter with the "Load" button. The Baton Manager gives access to a view that tells you who is logged on and who has current control of the beamline. This is

the panel the beamline team might ask you to open if they need to take control of the beamline. The Baton manager is available on other perspectives usually in association with the NCD Acquisition Buttons tab. The NCD Acquisition Buttons tab gives ready access to start/stop functions for NCD data collection together with control of the experiments hutch main shutter. This should not be confused with the fast shutter which is controlled through the TFG via a TTL signal (currently offline). Using the start button brings up a box in which you can save a title for your data collection. This is in text format so can be quite descriptive.

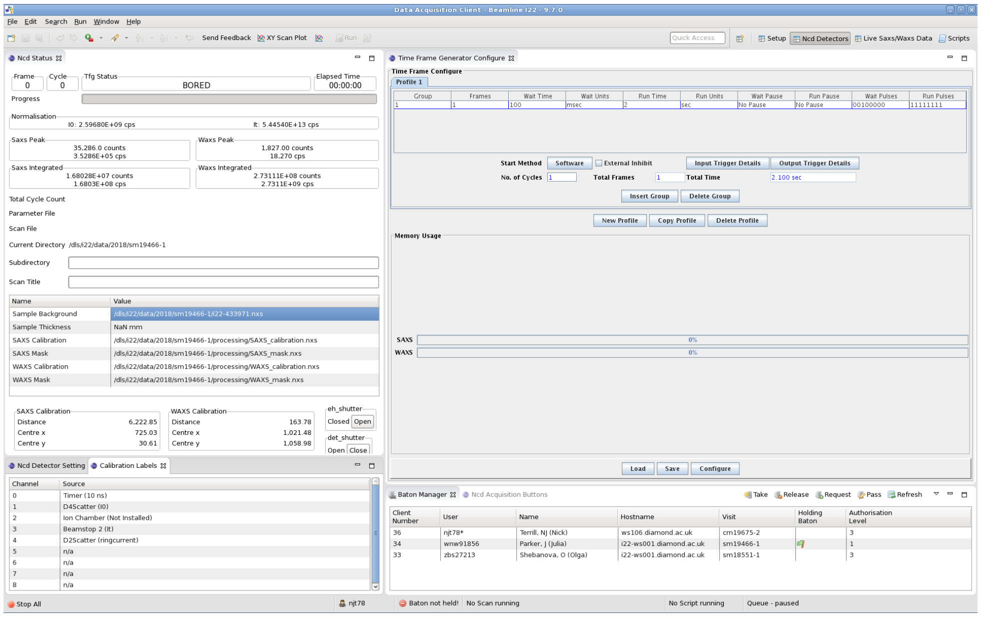

Fig. S3. NCD Detector Configuration Perspective

The Live SAXS/WAXS Perspective has been created to give users on I22 access to live SAXS and WAXS frames of the data being collected. For convenience the NCD Status, polling dashboard and Jython console tabs are repeated. Also included on this perspective is the SAXS and WAXS Data Source Panels. These panels allows you to select the source of the data to be displayed and to carry out some very simple manipulations on the data from the detector, addition/subtraction for comparison

purposes.

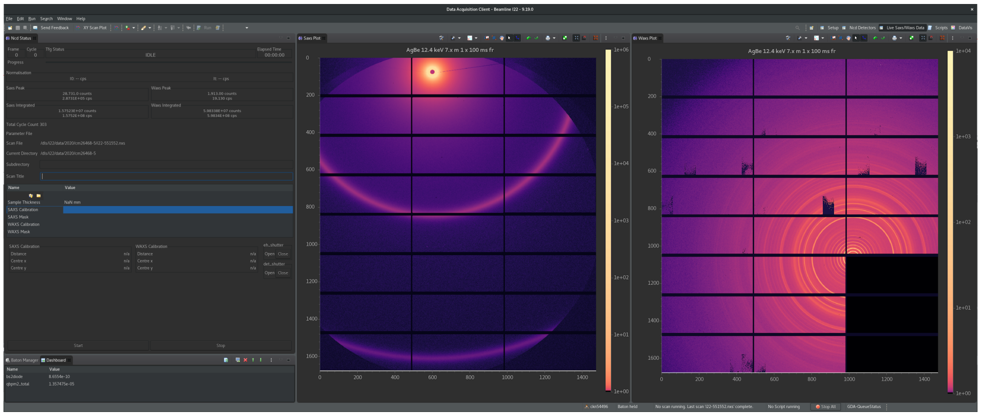

Fig. S4. Live SAXS/WAXS Perspective

3. Endstation and sample environments

The sample position on I22 is a very flexible space. Figure S5 shows a relatively complex setup with the P-jump pressure cell and a heated capillary rack, positioned side-by-side, to allow the user group to screen samples before conducting P-jump experiments.

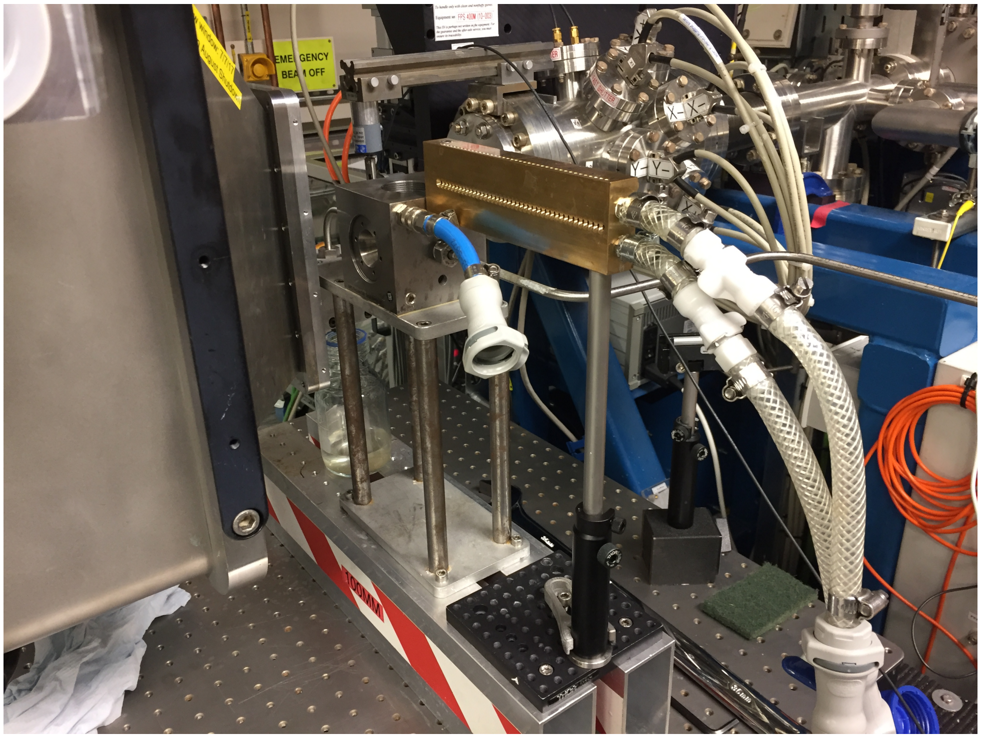

Fig. S5. P-jump cell and heated capillary rack mounted on the I22 sample platform
